# Expert consensus on standardized diagnosis and treatment for heat stroke

**DOI:** 10.1186/s40779-015-0056-z

**Published:** 2016-01-06

**Authors:** People’s Liberation Army Professional Committee of Critical Care Medicine

**Affiliations:** Department of Critical Care Medicine, Chinese PLA General Hospital, Beijing, China

**Keywords:** Sunstroke, Diagnosis, Cure, Medical treatment standards

## Abstract

Heat stroke is a life-threatening disease characterized clinically by central nervous system dysfunction and severe hyperthermia (core temperature rises to higher than 40 °C). The unchecked rise of body core temperature overwhelms intrinsic or extrinsic heat generation mechanism, thus overwhelms homoeostatic thermoregulation. Hyperthermia causes cellular and organ dysfunction with progressive exacerbation resulting in multi-organ failure and death. Rapid cooling to reduce core temperatureas quickly as possible is the primary and most effective treatment, as it has been shown that the major determinant of outcome in heatstroke is the degree and duration of hyperthermia. If suppression of body temperature is delayed, the fatality rate will be elevated. This is a guideline for the management of heat stroke, developed by the People’s Liberation Army Professional Committee of Critical Care Medicine lauched in June 2006. This is the first and origianl guideline for heat stroke in Chinese army and is expected to be properly used in daily clinial practice.

## Overview

Heats stroke (HS) is severe sunstroke caused by a rapid increase in one’s core temperature in excess of 40 °C from exposure to a hot and humid environment. HS is accompanied by serious clinical syndromes that damage multiple organ systems. These syndromes include burning skin and impaired awareness, such as delirium, convulsions, and loss of consciousness.

Exertional heat stroke (EHS) is caused by a rapid increase in one’s core temperature in excess of 40 °C from high intensity physical exercise in a hot and humid environment. EHS is accompanied by extremely serious clinical syndromes that damage multiple organs and multiple systems. Syndromes include impaired awareness, rhabdomyolysis, disseminated intravascular coagulation (DIC), acute liver damage, and acute renal damage. EHS is the most severe type of sunstroke and is characterized by acute onset and rapid progression The case fatality rate can reach more than 50 % if effective treatment is not received in a timely manner. EHS is commonly observed in healthy young people who exercise intensely in the summer, particularly soldiers and athletes who participate in summer training. As soon as participating soldiers are suspected of having EHS, they should be transferred immediately to a hospital for treatment.

Heat adaptation refers to a biological phenomenon in which people who live in hot environments for long periods significantly increase their heat tolerance capability compared with people who enter a hot environment for a short period of time. Heat adaptation is the result of several generations of adaptation established to maintain stability in and harmony with a hot climate. Heat adaptation is not only limited to physiological functions but is also known as biological heat adaptation because it is characterized by corresponding changes to the body’s external form and organ structure that have a stable, inheritable genetic foundation.

Acclimatization, an important concept in training and exercise physiology, refers to people adaptating to certain types of special environments.

Heat acclimatization, also known as acquired heat adaptation or physiological heat adaptation, is an acquired protective physiological reaction of the body to environmental heat stress. Heat acclimatization can be precipitated and strengthened or can decline and be lost. Heat acclimatization is the process by which people can reach a state of improved adaptation to a hot environment with certain theoretical guidance and medical monitoring.

Deacclimatization refers to the gradual weakening of heat tolerance and a return to its pre-acclimatization level once the heat stress effect ends.

## Epidemiological characteristics of heat stroke

### Heat stroke onset characteristics

Heat stroke onset is closely related to three environmental factors: high temperature, high humidity, and a windless environment.

Meteorological Threshold for Sunstroke: The threshold is a daily average temperature >30 °C or a relative humidity of >73 %. When excessive temperature and humidity conditions exist simultaneously, the rate of sunstroke occurrence increases significantly. There is a rapid and sudden increase in the number of sunstroke sufferers when the daily maximum temperature is ≥37 °C.

**Fig. 1:**
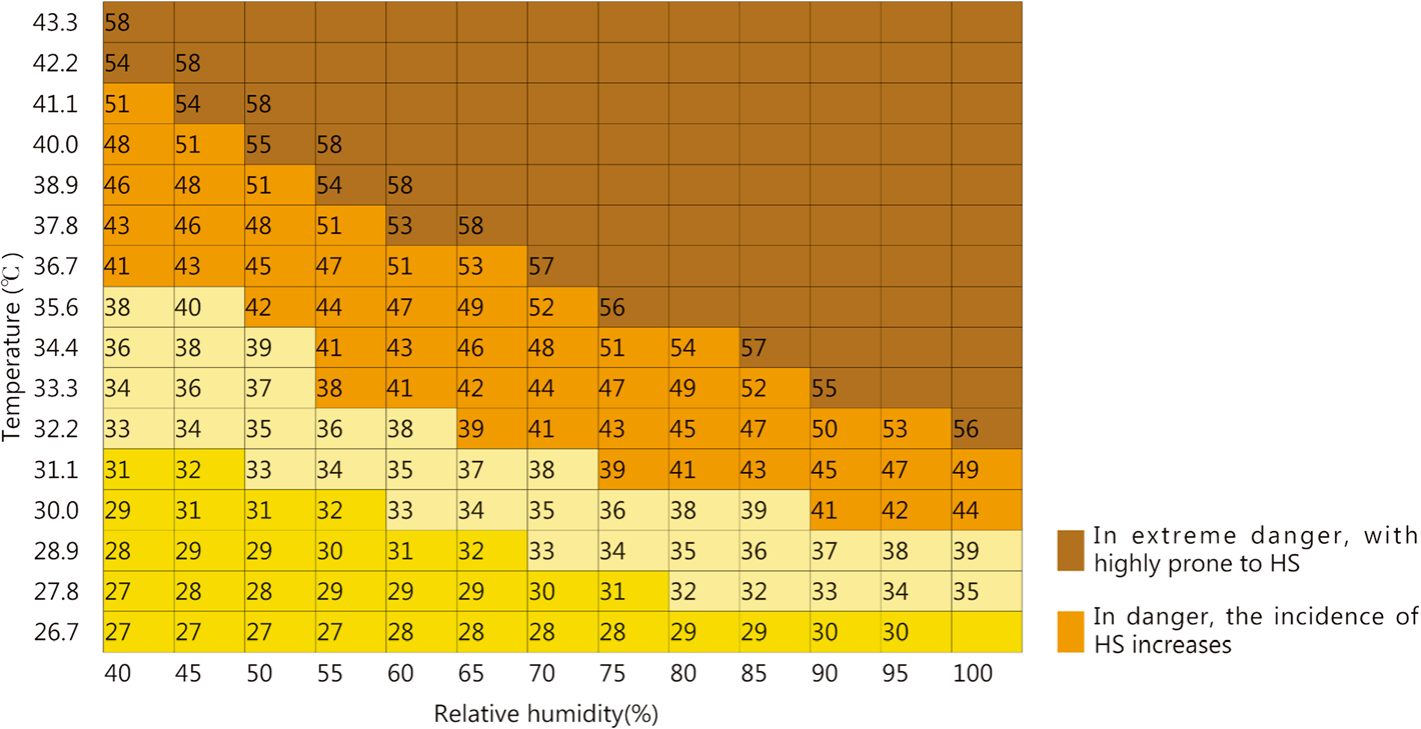
Heat index correlation with temperature and humidity

Heat Index: The heat index is a numerical value obtained by a mathematical operation using temperature and humidity levels. The heat index positively correlates with the rate of onset for heat stroke. When the heat index is >41, the heat stroke onset rate increases. When the heat index is >54, heat stroke is extremely likely to occur (Fig. 1).

### Predisposition factors

Individual factors are fever, common cold, gastroenteritis, diarrhea, vomiting, dehydration, sleep deficit, lack of heat acclimatization training, obesity, and hypokalemia.

Environmental factors are thermal overload on training grounds and strong direct sunlight

Organizational factors are training programs that are incompatible with physical capabilities, inadequate training and rest cycles, and inadequate water replenishment.

The overlay of predisposition factors increases the severity of heat stroke and correlates with the prognosis.

### Training intensity

The primary cause of EHS is the lack of heat acclimatization training for soldiers who undergo five-kilometer cross-country training during hot summer months.

## Clinical presentation

The clinical presentation of sunstrokes can be classified as pre-sunstroke, mild sunstroke, and severe sunstroke. Severe sunstrokes are further classified as heat cramps, heat exhaustion, and heat stroke (EHS and classic heat stroke).

### Pre-sunstroke

Pre-sunstroke occurs when headache, dizziness, thirst, sweating, sore and weak limbs, lack of focus and uncoordinated movement occur in a high-temperature environment. Body temperature may be normal or slightly elevated. If the patient is moved to a shady and breezy place in a timely manner to cool down and replenish water and salt intake, then recovery can occur within a short period.

### Mild sunstroke

In addition to the symptoms described above, mild sunstroke presents a body temperature that is often higher than 38 °C and may be accompanied by a ruddy complexion, excessive sweating, burning skin, the appearance of clammy and cold limbs, a pale complexion, falling blood pressure, and accelerated pulse rate. If the patient is moved to a shady and breezy place in a timely manner to lie down undressed, cool down, and replenish water and salt intake, then recovery can occur within several hours.

### Severe sunstroke

#### Heat cramps

Heat cramps are temporary, intermittent muscle spasms that may be associated with sodium loss. Heat cramps often happen to people who, upon initially entering a high-temperature work environment or during excessive physical exercise, sweat excessively and replenish only water intake.

Clinical Presentation: Heat cramps are temporary, intermittent muscle twitches that occur during or after training. Heat cramps can sometimes be confused with the twitching of the hands and feet caused by hyperventilation during heat exhaustion. The latter often presents with cramps in the hands and feet and numbness in the extremities and the perioral area.

Treatment Principle: Treatment is based on quickly moving the patient to a shady and breezy location to lie supine and replenish salt and water intake or drink electrolyte solutions that can readily relieve heat cramps. People suffering from mild symptoms can take oral rehydration salts. People who are dehydrated should receive an intravenous infusion of saline (0.9 % NaCl solution) and be actively prepared for transfer.

#### Heat exhaustion

Heat exhaustion refers to a group of clinical syndromes that are characterized by hypovolemia after heat stress. Under severe heat stress conditions, too much bodily fluid and sodium in the body are lost. An electrolyte imbalance occurs although no apparent damage to the central nervous system is presented.

Clinical Presentation: Clinical manifestations include sweating, fatigue, weakness, vertigo, headache, poor judgment, nausea, and vomiting. Sometimes muscle cramps, orthostatic dizziness, and fainting are also present. Body temperature is elevated although no apparent damage to the nervous system presents. Heat exhaustion can progress to heat stroke if not diagnosed and treated in a timely manner; therefore, heat exhaustion patients should immediately be sent to the hospital for treatment.

Laboratory Testing: Testing for elevated hematocrit, hypernatremia, mild azotemia, abnormal liver function, elevated creatine kinase (CK) should occur.

Treatment Principle: Rapid cooling and intravenous infusion are required when there is a severe reduction in blood volume or an electrolyte imbalance. If the blood pressure fluctuates with body position, then the patient should continue to be replenished with saline until hemodynamics are stabilized. The rest of the fluid loss can be supplemented slowly over a period of 48 h. Correction of hypernatremia that occurs too quickly can cause cerebral edema, leading to impaired awareness or epileptic seizures.

#### Heat stroke

The classic clinical manifestations of heat stroke are high fever, lack of sweat, and loss of consciousness. Because the cause of onset differs, clinical manifestations also differ.

##### Exertional heat stroke

EHS presents in healthy young people (such as soldiers who participate in training) who experience a sudden feeling of malaise after undergoing high intensity training or engaging in heavy physical labor for a period of time in a hot and humid environment. Fever, headache, dizziness, slow response, or sudden collapse and unconsciousness are accompanied by nausea, vomiting, shortness of breath, etc. A rapid increase in body temperature to 40 °C or higher follows, and delirium, lethargy, and loss of consciousness occur. The patient’s skin is hot and dry, and the complexion is ruddy or pale. The patient begins to sweat excessively or break out in a cold sweat followed by no sweat, tachycardia, shock, etc.

EHS is accompanied by severe rhabdomyolysis at the outset of a heat stroke. Therefore, acute renal failure, acute liver damage, and DIC appear early, emerging several hours to less than 20 h after onset. The patient’s condition deteriorates quickly, and the case fatality rate is extremely high.

Manifestations of Organ Function Damage from EHS:

1. Central Nervous System Damage. Serious nervous system dysfunction appears in the early stage characterized by restlessness, delirium, and loss of consciousness. Other neurological abnormalities may also appear, such as bizarre behavior, opisthotonus, hallucinations, decerebrate rigidity, and cerebellar dysfunction.
2. Coagulopathy. Clinical manifestations include skin bruising, bleeding and puncture site ecchymosis, conjunctival bleeding, black stools, bloody stools, hemoptysis, hematuria, myocardial hemorrhage, and intracranial hemorrhage. A combination of coagulopathy and DIC implies a poor prognosis.
3. Liver Dysfunction. Severe liver damage is an inherent characteristic of EHS. Aspartate aminotransferase (AST), alanine aminotransferase (ALT), and lactate dehydrogenase (LDH) increase rapidly after onset, reaching peak values in 3–4 days, then decrease gradually; elevated bilirubin lags behind, beginning to increase 24–27 h after the onset of a heat stroke.
4. Renal Dysfunction. Renal dysfunction is related to rhabdomyolysis. Manifestations are oliguria, anuria, and urine colored like dark tea or soy sauce. Acute oliguric renal failure appears in 25–30 % of EHS patients and 5 % of classic heat stroke patients.
5. Respiratory Dysfunction. Primary manifestations in the early stage are shortness of breath and cyanotic lips, developing into acute respiratory distress syndrome (ARDS).
6. Acute Gastrointestinal Dysfunction. Abdominal pain, diarrhea, watery stools, and gastrointestinal bleeding are commonly observed.
7. Cardiovascular Dysfunction. Hypovolemic shock manifests as hypotension, tachycardia (heart rate grater than 130 beats/min), and arrhythmia.
8. Rhabdomyolysis. Manifestations include muscle soreness and pain, stiffness, muscle weakness, tea-colored urine, and soy-sauce-colored urine. Muscle swelling and compartment syndrome may occur in the late stage.

##### Classic heat stroke

Class heat stroke is seen in elderly, frail, and chronically ill patients. Onset is generally gradual. Prodromal symptoms are difficult to identify. As symptoms become more serious after 1–2 days, blurred consciousness, delirium, and loss of consciousness occur. Incontinence and high body temperature up to 40–42 °C may appear. Heart failure and renal failure may also occur.

See Table 1 for the characteristics of EHS and classic heat stroke.

**Table 1:** Comparison of EHS and classic heat stroke characteristics

EHS	Classic
Healthy people	Existing predisposition factors/Taking medication
Young people	Middle-aged to elderly
Physical exercise	Sedentary
No sweat after sweating	Little sweating
Hypotension	Normal blood pressure
DIC	Mild coagulopathy
Rhabdomyolysis with apparent increase in CK	Slightly elevated CK
Acute renal failure	Oliguria
Significantly high lactic acidosis	Mild acidosis
Hypocalcemia	Normal serum calcium

## Laboratory testing

### Routine blood work

In the early stage of onset, blood concentration because of dehydration can appear in elevated hemoglobin (Hb) and increased hematocrit (Hct). Platelets (PLT) are normal during the initial stage of onset but then decrease rapidly. The decrease is particularly staggering 1–3 days after onset, down to a minimum of less than 10 × 10^9^/L.

### Infection indications

Increased white blood cells (WBC) and neutrophils indicate infection. Their levels of increase correlate with the severity of the sunstroke. Co-infection increases significantly and may be associated with elevated C-reactive protein (CRP), procalcitonin (PCT), and Interleukin-6 (IL-6).

### Blood biochemistry

Electrolytes: Hyperkalemia, hyponatremia, hypochloremia, hypocalcemia, and hyperphosphatemia.

Renal Function: Renal function shows varying degrees of elevated serum creatinine (Cr), blood urea nitrogen (BUN), and uric acid (UC).

Liver Function: Liver function shows significant increases in AST, ALT, and LDH in the early stage, up to 5,000 U/L or more. Total bilirubin (TBil) begins to increase after 24–72 h, up to 300 μmol/L or more, and may be associated with hypoalbuminemia.

Rhabdomyolysis: CK > 1,000 U/L, up to a maximum of 300,000-400,000 U/L, indicates rhabdomyolysis. CK > 5,000 U/L indicates severe muscle damage. CK > 16,000 U/L suggests a correlation with acute renal failure. Myoglobin (Mb) increases significantly. Typically, blood has Mb > 1,000 ng/ml, up to 70,000–80,000 ng/ml or higher; and urine has Mb > 500 ng/ml, up to 50,000 ng/ml or higher. Initial blood Mb is higher than urine Mb. As renal function is restored, the urine Mb becomes higher than the blood Mb.

### Coagulation

Coagulation dysfunction can appear on the first day of onset but is more commonly observed on the second or third day. Laboratory Testing Norms are ① PLT < 100 × 10^9^/L or progressive decrease; ② fibrinogen (Fib) < 1.5 g/L or progressive decrease; ③ D-2 aggregate increase or positive D-2 aggregate; fibrinogen degradation products (FDP) > 20 mg/L or 3P test positive; ④ prothrombin time (PT) extension to longer than 3 s; and activated partial thromboplastin time (APTT) extension to longer than 10 s. If someone suffers from 3 of the aforementioned abnormalities, a diagnosis of DIC can be rendered. Coagulation should be rechecked every 4–6 h during the early stage of onset. If conditions are feasible, thrombelastograph (TEG) and the coagulation and platelet function analyzer (Sonoclot) can be used in the examination.

### Arterial blood gas

Arterial blood gas often refers to metabolic acidosis and respiratory alkalosis, lactic acidosis, hypoxemia, etc.

### Routine urine testing and urine biochemistry

Microscopic examination of tea- or soy-sauce-colored urine shows a large amount of granular casts and red blood cells and an increase in Mb.

### Routine fecal testing

Fecal occult blood can be positive.

### Electrocardiogram

Electrocardiograms show more tachyarrhythmia. This is generally sinus tachycardia from premature ventricular contraction; electrocardiograms can also occasionally show bradycardia and may be associated with abnormal T waves and ST segments.

### Cranial Computerized Tomography (CT) examination

There are scarcely any positive CT findings during the early stage of onset. After 3–5 days, diffused parenchymal brain edema may appear. Coagulation dysfunction sufferers may show subarachnoid hemorrhage.

### Cranial Magnetic Resonance Imaging (MRI) examination

MRI during the late stage of heat stroke shows ischemia and malacia in the basal ganglia, globus pallidus, bilateral internal capsule, putamen, and cerebellum. Some patients’ MRIs indicate abnormalities in the bilateral cerebellum, caudate nucleus, and subcortical white matter and an even increase in the hippocampus. In severe cases, cerebellar ischemic necrosis or even brain atrophy occurs.

## Diagnosis

Patients exposed to a hot and humid environment to engage in high intensity exercise may also have the following clinical presentation: severe manifestations of central nervous system dysfunction (such as loss of consciousness, convulsions, and delirium), a core temperature above 40 °C, elevated skin temperature and/or continual sweating, significant increase in liver transaminases significant decrease in platelets and rapid appearance of DIC, muscle weakness, pain, tea-colored urine, and CK greater than 5 times the normal value.

## Treatment

Early effective treatment is the key to determining the prognosis. The crucial points in effective treatment are rapid lowering of the core temperature, blood purification, and DIC prevention.

Specific treatment measures are “nine early and one ban,” that is, early cooling, early expansion, early blood purification, early sedation, early intubation, early correction of coagulation dysfunction, early resistance to infection, early enteral nutrition, early immunoregulation, and a ban on surgical operations during the period of coagulation dysfunction.

### Cooling

Rapid cooling is the most important treatment measure. The case fatality rate is closely related to hyperthermia and its duration. If cooling is delayed, the fatality rate increases significantly. As soon as a patient is removed from the hot environment, immediately begin cooling and continue to monitor core temperature. Cooling targets are to quickly cool the core temperature to 39 °C or below within 10–40 min and to 38.5 °C or below within 2 h.

#### On-site cooling

① Quickly move the patient from a hot and humid environment to a shady and breezy place, make the patient lie down, and remove all clothing; ② use a cold water spray or wet towels to wipe the entire body; ③ use fanning to accelerate evaporation and convection cooling; ④ continue to monitor body temperature.

#### Cooling en route

① Turn on the air conditioning in the ambulance or open the windows; ② use cold water to wipe the entire body; ③ and administer an intravenous infusion. Continue to monitor body temperature.

#### Cooling in the sickroom

① Adjust the room temperature to 20–24 °C, ② administer an intravenous infusion quickly, ③ use cooling blankets, ④ place ice cubes on areas that dissipate heat faster (on either side of the neck, groin, and armpits), ⑤ use 200–500 ml of 4 °C saline to perform gastric lavage and/or rectal enema, ⑥ purify the blood, ⑦ use a lytic cocktail in combination, and ⑧ if conditions allow, use an intravascular cooling apparatus or immerse the patient in a cold water bath (water temperature at 15–20 °C).

### Circulation monitoring and fluid resuscitation

Circulation Monitoring: Continuously monitor blood pressure, heart rate, respiratory rate, pulse oximetry (SPO_2_), blood, and hourly urine output and urine color; and monitor central venous pressure (CVP) as needed. For fluid resuscitation, first, select the crystalloid solution—such as saline, glucose solution, or Ringer solution—then control the infusion rate to maintain urine output at 200–300 ml/h. ② In the case of sufficient urine output, the total infusion amount in the first 24 h can be up to approximately 6–10 L. Adjust the infusion rate based on the dynamic monitoring of blood pressure, pulse, and urine output. A diuretic may be necessary: if the urine output does not yet meet the target after adequate rehydration expansion, administer an intravenous bolus with 10–20 mg furosemide with a follow-up dose depending on urine output. Simultaneously, take care to monitor electrolytes and replenish potassium in a timely manner; ④ for alkaline urine, supplement with sodium bicarbonate so that the urine has pH > 6.5.

### Blood purification

A patient who has one of the following conditions may be considered for continuous bedside continuous renal replacement therapy (CRRT). Patients with two or more of the following conditions should undergo hemofiltration treatment immediately: ① typical physical cooling methods are ineffective and body temperature continues to be higher than 40 °C for more than 2 h; ② serum potassium > 6.5 mmol/L; ③ CK > 5,000 U/L or the rate of increase exceeds 1 fold/12 h; ④ oliguria, anuria, or difficulty in controlling volume overload; ⑤ Cr daily incremental increase value > 44.2 μmol/L; ⑥ difficulty in correcting the electrolyte and acid–base imbalance; ⑦ instability in hemodynamics; ⑧ severe infection, sepsis; ⑨ a combination of multiple organ damage or the appearance of multiple organ dysfunction syndrome (MODS).

Indications for CRRT Termination: ① stability in vital signs and the patient’s condition; ② CK < 1,000 U/L; ③ correction in the water, electrolyte, and acid–base imbalance; ④ urine output > 1500 ml/day or resumption of normal renal function.

Hemodialysis or peritoneal dialysis may be considered as maintenance treatment for patients whose renal function cannot return to normal but whose other organs have all resumed normal function.

### Sedation and analgesia

Restlessness and twitching may appear in heat stroke patients. Choose sedatives with fast efficacy, strong effectiveness, and few side effects such as propofol and benzodiazepines. The following are gradations of treatment measures.

#### On-site treatment

Intramuscular injection with 10–20 mg Valium.

#### Basic hospital treatment

① Begin with an intravenous injection of 10–20 mg Valium dispensed within 2–3 min. If an intravenous injection is difficult, an intramuscular injection can be administered immediately. If the initial drug does not control the twitching, follow up with a 10 mg intravenous injection after 20 min. The total amount of drugs should not exceed 40–50 mg in 24 h: ② an intravenous injection of 12.5-25.0 mg chlorpromazine and ③ an intravenous injection of 12.5-25.0 mg promethazine.

#### Central hospital treatment

(1) Propofol: 0.3-0.6 mg/(kg^.^h) by injection pump for adults; (2) midazolam (imidazole valium): first, intravenous injection 2–3 mg for adults, then 0.05-0.10 mg/(kg^.^h) by injection pump; (3) analgesia: meperidine, a single intramuscular injection of 50–100 mg with a maximum daily dose of 200 mg; morphine, a single intramuscular injection of 5–10 mg with a maximum daily dose of 20 mg; fentanyl, 0.6 μg/(kg^.^h) by injection pump with a maximum daily dose of 0.3 mg.

One must monitor drug dosage, infusion rate, and patient response. In an overdose, pay attention to the occurrence of respiratory depression and low blood pressure.

### Intubation indications

Intubation indications are (1) impaired awareness, (2) secretions in the airway and inability to expectorate on one’s own, (3) aspiration, (4) deep sedation, (5) respiratory failure with PaO_2_ < 60 mmHg and progressive deterioration in oxygenation, and (6) unstable hemodynamics and poor response to fluid resuscitation and vasoactive drugs.

### Correction of blood dysfunction

The correction of blood dysfunction primarily includes the replenishment of coagulation factors followed by anticoagulation therapy.

#### Replenishment of coagulation factors

Coagulation factors (such as fresh frozen plasma, prothrombin complex, fibrinogen, cryoprecipitate, etc.) should be replenished as soon as possible. ① For fresh frozen plasma, the first dose is 10–15 ml/kg, followed by the addition of 200–400 ml according to the coagulation indices being monitored. Restore PT and APTT to normal levels. ② For cryoprecipitate, use 5–10 U/each time.

#### Replenishment of platelets

If platelets are <50 × 10^9^/L, then the patient can be infused with 1 therapeutic dose of apheresis platelets. One unit of platelets can theoretically raise the platelets by (10–20) × 10^9^/L. Assess treatment effectiveness by rechecking platelet counts 1 h after infusion.

#### Anticoagulation

##### Anticoagulation opportunity

D-2 aggregate increases significantly after active replenishment of coagulation factors. Anticoagulation treatment should be administered in the early stage. Take care to monitor coagulation correlation indices such as PT, APTT, international normalized ratio (INR), Fib, and D-2 aggregate.

##### Commonly used anticoagulants and dosage

1. For low molecular weight heparin, the total daily amount is 100–200 U/kg, divided into 2 subcutaneous injections, 1 time/12 h.
2. For unfractionated heparin, advocate the clinical use of a micro-pump to administer the drug intravenously; the total daily amount is 1.5-3.0 mg/kg.
  Terminate or temporarily suspend the use of anticoagulants if active bleeding occurs (such as intracranial hemorrhage, gastrointestinal bleeding, etc.) and the amount of bleeding is relatively substantial (a daily infusion of 2 units of red blood cells may be required to maintain the patient’s Hb).
3. Timing of Medication Withdrawal: Continue with the course of treatment until PLT can be maintained at a desired level. Medication can be stopped when all coagulation indices, such as D-2 aggregate, maintain normal levels for 1 week or longer. After medication withdrawal, monitor changes in coagulation weekly for 2–3 weeks. Individual patients whose D-2 aggregate is elevated again after medication withdrawal require a new course of anticoagulants.

### Resistance to infection

Infection can be resisted in the early stage by the prophylactic use of antibiotics such as second generation cephalosporin antibiotics. If there is infection, collect relevant specimens for smears and culture in a timely manner, increase the level of antibiotics, and add anti-fungals as necessary.

### Enteral nutrition

If the patient’s hemodynamics and internal environment are stable and there is no gastrointestinal bleeding and paralytic ileus, enteral nutrition should be administered as soon as possible.

#### Application principle

① Choose a feeding tube path (nasogastric/nasoenteric) for patients who cannot take food by mouth to establish a manner with which to support enteral nutrition. ② Elevate the head of the patient who uses a nasogastric/nasoenteric feeding tube during feeding time by 30–45° to reduce the occurrence of aspiration pneumonia.

#### Infusion method

To ensure the safe input of enteral nutrition preparations, the enteral nutrition infusion method should be determined according to the patient’s condition, recipe type, and input pathway. Enteral nutrition infusion should follow the principle of gradual progression from a small amount of nutrition to a greater amount, from slow to fast, and from thin to more concentrated. The temperature should be maintained at 37–40 °C.

The use of a nasal feeding pump for continuous enteral nutrition infusion generally begins at 20 ml/h. If the patient can tolerate it, the rate can gradually be increased; for those who cannot tolerate the infusion, the rate can be lowered to a tolerable level and then gradually increased again.

#### Choice of enteral nutrition preparations

Choose different enteral nutrition preparations according to the patient’s level of liver and renal dysfunction. The preparations can be classified as short peptide preparation and whole protein homogenized meal. When choosing enteral nutrition preparations for patients suffering from gastrointestinal dysfunction, one must begin with the short peptide preparation and gradually move to the entire protein homogenized meal. When the patient’s condition is critical, a low-calorie intake of 20–25 kcal/(kg^.^d) is permissible.

#### Precautions

Notably, when providing enteral nutrition via a nasogastric path, regular retrieval of stomach content is necessary to assess whether there is gastric retention so that timely adjustments can be made to the rate and total amount of infusion. Observe for abdominal distention, diarrhea, and other negative reactions. If the patient has abdominal distention and the abdominal pain intensifies, particularly when abdominal pressure increases, then enteral nutrition must stop.

### Anti-inflammation and immunoregulation

#### Ulinastatin

Ulinastatin has significant anti-inflammatory and immunoregulatory effects and can reduce the systemic inflammatory response and protect organ function. The recommended dose is 400,000–800,000 U, 2 times/day, for a 7–10 day course of treatment.

#### Glucocorticoids

(1) Patients who conform to one of the following can be considered for glucocorticoid use: ① persistent fever ≥39 °C accompanied by multiple or large consolidation and/or a shadow in lung radiography with rapid progress within a short period; ② significant breathing distress sufficiently severe to meet the criteria for a diagnosis of ARDS. (2) Usage: The recommended dose for adults is dexamethasone 7.5 mg/day or hydrocortisone 200 mg/day or methylprednisolone 80–120 mg/day by intravenous infusion, adjusted according to the patient’s condition and individual differences. (3) Simultaneously, administer antacids and mucosal protective agents, monitor and control blood sugar to 8–10 mmol/L, and prevent double infection.

#### Thymosin and gamma globulin

Based on the patient’s condition, apply 1.6 mg thymosin, 1 time/day or every other day, for a 7–10 day course of treatment or apply gamma globulin 10 g/day for a 7–10 day course of treatment.

### Ban on surgery in the early stage and other unnecessary invasive procedures

Because heat stroke patients in the early stage often also have coagulopathy, such patients are prone to DIC. Surgical and other invasive procedures tend to increase bleeding, which can even be life-threatening. Therefore, with the exception of necessary operations such as blood purification catheter insertion, central venous catheter insertion, etc., surgical procedures should be minimized (such as tracheotomy and decompression surgery by fascia cavity incision) as much as possible.

## Prognosis

Factors that affect prognosis include ① fever duration, ② cooling rate, and ③ degree of damage to the body, including severe coagulopathy, acute renal failure, metabolic acidosis, elevated CK > 10,000 U/L, and elevated liver enzymes >3,000 U/L. The case fatality rate increases significantly for patients who have two or more of the aforementioned factors. In addition, ④ central nervous system factors affect the case fatality rate, including loss of consciousness and its duration. Despite the rapid cooling treatment administered, individual patients may nevertheless recover from heat stroke with permanent neuropsychiatric sequelae.

## Prevention

### Heat acclimatization implementation

Heat acclimatization training is an effective measure for sunstroke. This process takes 10–14 days. Heat acclimatization training for troops should be organized before troops from a cold zone or warm zone are stationed in a hot zone or before troops from a hot zone begin annual high-intensity training in early summer.

#### Temperature adaptation

The ambient temperature during training should go from low to high. Extreme hot weather should be avoided during the initial phase of training. An initial temperature of 30 °C is appropriate. Transition gradually each day to train during a hotter portion of the day with temperatures at 31–37 °C.

#### Intensity adaptation

Only training with sufficient intensity within physiological tolerance limits can help troops attain a high level of heat acclimatization and the ability to complete high-intensity training. During implementation, the amount of physical exercise should go from small to large with a gradual increase in exercise intensity. Marching, march load, ball-playing or other training or physical exercise that can improve the endurance of the cardiovascular system can be interspersed. In hot weather conditions, cross-country and marathon training are beneficial; the results of combined cross-country and marching training are even better.

#### Appropriate training period

The duration of heat acclimatization training is best at 1.5-2.0 h (no less than 50 min) during the initial phase. The method for monitoring training intensity and physiological limits has several components. To understand physiological tolerance levels, each participant should take his own pulse for half a minute at the end of each training session as timed by an oral order from the military doctor. Each training should comprise 1–2 sessions. The training period should be 1–2 weeks. The total number of training sessions cannot be less than 6–12 times; otherwise, the troops cannot acclimate well to heat.

#### Repeated strengthening and improvement of heat acclimatization results

Training should continue after attaining heat acclimatization. Consolidation training should occur no less than 2–3 times per week to continually strengthen and improve heat acclimatization levels. If training is interrupted or the trainees leave the hot environment, then deacclimatization occurs.

#### Training termination

Training should be terminated when trainees look pale and walk with a drunken gait during training and when the training intensity is shown to have exceeded the upper limit of human tolerance (heart rate > 170 beats/min, body temperature exceeds 39 °C). The trainees can participate in training again after these values return to normal.

#### Deacclimatization

The rate of deacclimatization differs according to one’s acclimatization level and individual health conditions. The decline in cardiovascular acclimatization capability is more pronounced and faster than the decline in body temperature acclimatization capability. Deacclimatization occurs 1–2 weeks after the termination of heat acclimatization training. After deacclimatization, renewed training can attain heat acclimatization in a shorter period of time than was originally required.

### Improvement of relevant safeguards

Dehydration, lack of salt, overtraining, sleep deficit, nutritional deficiency, inadequate caloric intake, etc., can delay heat acclimatization and should be prevented from occuring during training.

#### Reasonable diet and water and salt replenishment

A spicy, high-fat diet heavy in meat and fish is inappropriate in the summer. Logistics must ensure a supply of sunstroke prevention beverages such as cold salt water, cold water, and mung bean soup. Water is an important “tactical weapon” in sunstroke prevention. Drink sufficient water before marching, training, or work; fill canteens; replenish the body with 2 l of water every 4 h (approximately 2 military canteens); at midday, replenish with 1 l of water every 1.0-1.5 h (approximately 1 military canteen); or adjust the amount of water intake as appropriate in accordance with the temperature, activity intensity, and the amount of sweat. Drinking water at a temperature of 8–12 °C is preferred. Natural water temperature is also suitable.

The amount of water consumed based on thirst alone is insufficient to maintain fluid balance. Excessive water intake is good; that is, as much as possible, drink more than the amount of water necessary to satisfy thirst each time. Water consumption up to 70 % of the amount of sweat can more efficiently improve physiological functions when one is laboring at high temperatures and prevent the occurrence of heat stroke. However, in the case of excessive sweating (6 l daily), excessive water intake places too much burden on the gastrointestinal tract (bloating) and will likely cause fatigue. In short, one should drink small amounts of water and drink many times; binge drinking is inappropriate. Sufficient fluid intake helps to avoid placing more burden on the heart and the gastrointestinal tract and reflexively causing more sweating and more water and salt excretion through the kidneys. One should place importance on the replenishment of salts while replenishing water. The daily need for salts can typically be supplemented by diet. Soup should be served at each meal, and the soup dish can be slightly saltier than normal. Oral rehydration salts can be carried during long periods of field marching and added to drinking water before consumption.

#### Necessary sleep and rest guarantee

Summer has long days and short nights with high temperatures. The body’s metabolism is vigorous. High temperatures combined with high-intensity training or labor cause the body to feel tired. Adequate sleep allows the brain and all body systems to relax; sleep is an essential measure for heat stroke prevention. Therefore, training time should be formulated scientifically. One should avoid strong sunlight and periods of higher temperatureand shorten or reduce continuous training time in the hot sun or in a high-temperature environment. Reasonable arrangements should be made for rest and appropriately lengthened lunch breaks. If such circumstances are not possible because of mission requirements, appropriate protective measures must be taken.

#### Formulation of an individualized training program based on individual physical condition

Soldiers who have recently suffered from sunstroke, the common cold, fever, abdominal pain, diarrhea, overload, too little sleep from being on night duty, or new soldiers, etc., should be classified as key observation targets and monitored appropriately. Health personnel must thoroughly understand the platoon, the squad, and the site. Health personnel must focus on heat-stroke-prone environments and targets, strengthen medical supervision, and identify and address problems in a timely fashion.

## Evacuation

Once a soldier participating in training is suspected of having a heat stroke, he should be immediately transferred to a hospital in the rear for treatment.

### Evacuation indications

① The patient’s body temperature is >40 °C, ② the patient’s body temperature remains at >40 °C after cooling measures are taken (the patient is carried to a shady and cool place, sprinkled with water, and fanned continually for 15 min), ③ there is no improvement in the patient’s impaired awareness, and ④ there is a lack of necessary conditions for treatment.

### Pre-transfer contact

Inform the medical unit in the rear of the patient’s condition, required equipment, medication for subsequent treatment, and the patient’s estimated arrival time.

### Pre-transfer preparation

① The ambulance should be equipped with air conditioning. If there is no air conditioning, the windows of the ambulance should be able to be opened. ② Transfer personnel generally include 1 doctor and 1 nurse. ③ Accompanying equipment includes thermometers, sphygmomanometers, stethoscopes, pulse oximeter, electrocardiographic monitor, etc., or monitors that provide information on the the aforementioned vital signs. Airway management devices include endotracheal intubation equipment, laryngeal mask, mask, oropharyngeal airway, portable suction equipment, and portable artificial respiration equipment. Oxygen supply equipment must meet the patient’s oxygen supply needs during the entire transport journey in addition to a minimum of 30 min of spare oxygen. The ambulance should be equipped with a defibrillator as necessary. ④ Accompanying medication should include epinephrine, dopamine, norepinephrine, and antiarrhythmic drugs such as lidocaine and amiodarone; pharmaceuticals such as Valium, midazolam (imidazole valium), propofol, meperidine, morphine, and fentanyl; and sufficient amounts of saline or balanced salt solution.

### Body temperature monitoring and treatment during transit

#### Body temperature monitoring en route

Body temperature should be closely monitored during transit and measured once every 0.5-1.0 h.

#### Commonly used cooling methods

① Turn the air conditioning in the ambulance to the lowest temperature setting, or open the vehicle windows. ② Use warm water at 15–20 °C (obtained from the nearest source—well water, spring water, river water, etc.) to repeatedly wipe the entire body to promote cooling in conjunction with continuous fanning. If there is ice, it can be used to cool the head area. Ice can also be used to cool areas with large blood vessels such as the armpits and the groin area. ③ Conscious patients can orally take 4–10 °C saline or 500–1,000 ml Ringer solution.

### Monitoring and life support during transit

Continue with electrocardiographic monitoring of arterial blood pressure, heart rate, respiration, pulse oximetry (SpO2), and electrocardiogram during transit.

#### Oxygen inhalation

Use an oxygen mask or nasal cannula for oxygen inhalation. The oxygen flow rate is 3–5 L/min. Continuously monitor SpO_2,_ and maintain SpO_2_ above 90 %.

#### Airway management

① For regurgitation management, as soon as vomiting occurs in the patient, immediately turn the patient’s head to the side; clear the mouth and nasal passage of secretions in a timely manner and keep the airway open to avoid aspiration. ② If there is choking, after suctioning out secretions and vomit from the mouth and nasal passage, use the head-chin lift method to tilt the patient’s head back and pull the jaw down to open the patient’s airway. Oropharyngeal airway or endotracheal intubation can be inserted in patients who qualify for this treatment. When necessary, a thick needle can be used to perform an emergency cricothyroid membrane puncture or tracheotomy incision. ③ For twitching, the treatment is an intravenous infusion with 10 mg diazepam (Valium). If this treatment is ineffective, another 10 mg can be administered after 5–10 min until twitching is under control. Care must be taken to prevent the patient from biting his tongue and to keep the airway open.

### Contact during transit

If the patient’s condition changes during transit, contact the hospital in the rear for treatment assistance at any time. Contact the hospital in the rear 30 min before expected arrival, and be prepared to provide support.

### Transport to treatment unit at the hospital in the rear

Follow-up treatment can proceed in a timely fashion through doctor-doctor and nurse-nurse handover. The transfer contents include the patient’s condition, the state of the patient during the entire transport process, and the treatment plan.

